# Changes in Diversification Patterns and Signatures of Selection during the Evolution of Murinae-Associated Hantaviruses

**DOI:** 10.3390/v6031112

**Published:** 2014-03-10

**Authors:** Guillaume Castel, Maria Razzauti, Emmanuelle Jousselin, Gael J. Kergoat, Jean-François Cosson

**Affiliations:** INRA, UMR 1062 CBGP (INRA, IRD, Cirad, Montpellier SupAgro), Campus de Baillarguet, 34988 Montferrier-sur-Lez, France; E-Mails: maria.razzauti@supagro.inra.fr (M.R.); jousseli@supagro.inra.fr (E.J.); kergoat@supgro.inra.fr (G.J.K.); jean-francois.cosson@supagro.inra.fr (J.F.C.)

**Keywords:** diversification rate, Hantavirus, molecular evolution, Murinae, phylogenetics, selection

## Abstract

In the last 50 years, hantaviruses have significantly affected public health worldwide, but the exact extent of the distribution of hantavirus diseases, species and lineages and the risk of their emergence into new geographic areas are still poorly known. In particular, the determinants of molecular evolution of hantaviruses circulating in different geographical areas or different host species are poorly documented. Yet, this understanding is essential for the establishment of more accurate scenarios of hantavirus emergence under different climatic and environmental constraints. In this study, we focused on Murinae-associated hantaviruses (mainly Seoul Dobrava and Hantaan virus) using sequences available in GenBank and conducted several complementary phylogenetic inferences. We sought for signatures of selection and changes in patterns and rates of diversification in order to characterize hantaviruses’ molecular evolution at different geographical scales (global and local). We then investigated whether these events were localized in particular geographic areas. Our phylogenetic analyses supported the assumption that RNA virus molecular variations were under strong evolutionary constraints and revealed changes in patterns of diversification during the evolutionary history of hantaviruses. These analyses provide new knowledge on the molecular evolution of hantaviruses at different scales of time and space.

## 1. Introduction

Pathologies caused by hantaviruses are considered as emerging diseases with important public health impact worldwide because of the recent detection of new species of hantavirus in Africa, Asia, America and Europe [1,2,3,4,5], the increase in the amplitude and frequency of outbreaks of human hantaviruses [6,7,8] and the expansion of the geographic distribution of Seoul virus in the United Kingdom, Belgium and France [2,9,10]. The importance of hantaviral diseases is still strongly underestimated due to the lack of surveillance in many countries, the non-census of a large majority of benign cases and poor knowledge on wildlife reservoirs. Recently, various studies have shown that interactions between wild reservoirs and hantaviruses were more complex than previously described. The identification of reservoir species, their specificity with hantaviruses species and the long coevolution between hantaviruses and rodents have been particularly challenged by the discovery of hantaviruses in soricomorphes and bats [11,12,13,14], by co-phylogenetic studies [14,15], and by the occurrence of many host shift and overlapping host ranges [14,16,17,18]. In humans, hantaviruses are responsible of the Hantavirus Cardio-Pulmonary Syndrome (HCPS) in the Western hemisphere and the Haemorrhagic Fever with Renal Syndrome (HFRS) in the Old Word [8]. An outbreak of HCPS occurred in the summer of 2012 in Yosemite National Park in California with a high rate of fatal cases (30%) [7] and about 150,000 cases of HFRS are thought to occur annually worldwide [8].

A better understanding of mechanisms that drive and control viral genetic variation may help us to predict and prevent scenarios that lead to the emergence of viral diseases [19]. Hantavirus molecular variation has long been overlooked, being mostly restricted to phylogenetic studies and species descriptions. However, the scientific community currently exhibits a renewed interest in understanding lineage diversification and molecular factors favouring host switching and pathogenic severity towards humans. Hantaviruses are ssRNA viruses with a genome organization typical of members of the Bunyaviridae, consisting of three negative-stranded RNA segments: a large L segment encoding the viral RNA polymerase, a medium M segment encoding the envelope glycoproteins Gn and Gc, and a small S segment encoding the viral nucleocapsid protein. Reassortments (*i.e*., exchange of genome segments) can occur between different strains or even between different species when they infect the same host cell [18]. As other RNA viruses, hantaviruses have a high replication rate and a poor proof-reading ability of their RNA-dependent RNA polymerase but it is now known that evolutionary rates in these viruses are explained by many different aspects of viral biology and not only by polymerase fidelity [20]. Host switching and introduction into new environments can also promote viral evolution [21,22]. Because of their small size, important biological functions are compactly integrated in viral genomes and a limited number of mutations/genomes can be sufficient to drive a virus to extinction. Functional conservation of the viral proteins is reflected by purifying selection with the exception of those involved in evading the host immune system [21,22].

Hantaviruses exist within their host as complex and heterogeneous populations [23,24] and genetic drift, neutral mutation accumulation and small deletion/insertion within the non-coding regions of RNA segments seem to be the main mechanisms involved in promoting their genetic diversity. However, the genetic diversity measured at a given time point does not seem to be translated into a high rate of accumulation of genetic changes on longer time-scales [25]. A recent study on Puumala virus (PUUV) molecular variation in voles described a high rate of apparition of transient variants, including reassortants, but also the preservation of a few preferred genotypes over time [26]. Yet it is not clear whether this pattern better supports a neutral mode of microevolution mostly driven by genetic drift rather than molecular conservatism due to negative selection. Recent studies actually provided evidence of the molecular signature of negative selection in wild-type Murinae-associated hantaviruses, a phenomenon that may be reversed by a treatment using a mutagen-increasing molecule, such as ribavirin, which induces increased mutational load and positive selection [27,28,29]. In any case, this dynamics is corroborated by a slow rate of nucleotides change over time while, as other RNA viruses, hantaviruses exhibit very high short-term substitution rates. 

Different modes of ssRNA virus evolution have recently been described by Holmes [30,31]. The process of lineage diversification primarily occurs when viruses infect new host species, either through cross-species transmission or codivergence, (*i.e*., the process of parallel cladogenesis in which the diversification of one biological entity results in the diversification of its associated biological entities [32]). Alternatively, it can be driven by the acquisition of new niches within the same host species; this event is defined as intrahost duplication. In a recent study, Kitchen *et al.* have tested these two alternative evolutionary scenarios in ssRNA viruses from five families of RNA viruses [33]. They concluded that the colonization of new but related host species might represent the main mode of diversification in RNA viruses, although strong biases in our knowledge of viral biodiversity may actually blur the actual pattern. Until recently, hantavirus evolution was still seen as the result of tight coevolution with their rodent hosts but this assumption was challenged by Ramsden *et al.* [15] who proposed that there was no co-divergence between hantaviruses and their hosts. The parallelism between hantaviruses and hosts phylogenies could have been the result of the recent colonization of rodents by hantaviruses followed by shifts toward different host species (a phenomenon referred to as phylogenetic tracking) [34]. However, there is still great uncertainty regarding the history and timescale of the evolution of hantaviruses [14], which impacts our ability to predict the likelihood of future host jumps. Moreover, the determinants of diversification rate variability among closely related viruses or among lineages of the same viral species circulating in different geographic region or host species are still poorly understood [35]. As for other RNA viruses [36], environmental factors could have played an important role in hantavirus diversification. 

In this study, we focused on Murinae-associated hantaviruses and attempted to explore their mode of diversification at both global and local geographic scales. Eleven hantaviruses species and major lineages (whose specific statuses are still under debate) are carried by murine rodents: Haantan virus (HTNV) widely distributed in eastern Asia, together with Dabieshan virus (DBSV) [37], Amur virus (AMRV) [38] and Soochong virus (SOOV) [39]; Thailand virus (THAIV) [40], Serang virus (SERV) [41] and Jurong virus [42] in Southeast Asia; GOU virus (GOUV) [37]) in China; Sangassou virus (SANGV) [43] in Africa; Dobrava-Belgrade virus (DOBV) including four genotypes (Dobrava, Saarema (SAAV), Sochi and Kurkino) [44,45] in Europe; and Seoul virus (SEOV) worldwide. To this aim, we used sequences available from GenBank to conduct several phylogenetically-based approaches and investigate selection and shifts in patterns of lineage diversification. We then searched if these events characterized strains that are associated with specific geographic areas.

## 2. Results

### 2.1. Phylogenetic Analyses

**Figure 1:**
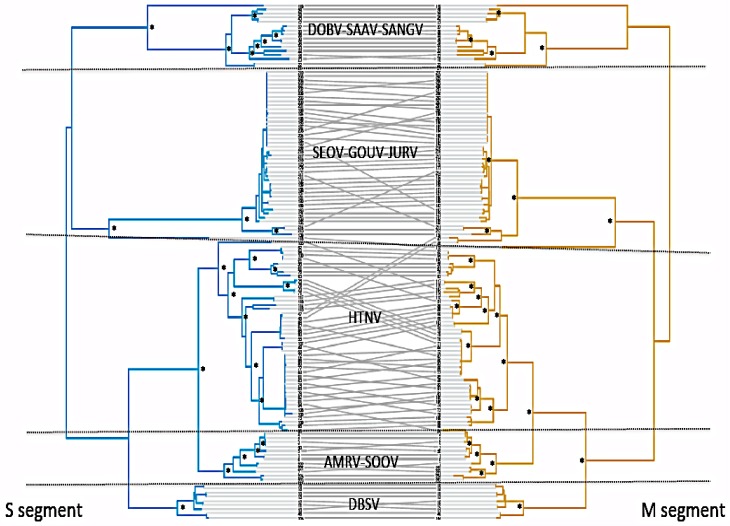
Tanglegram of the two phylogenetic trees based on analyses of segments M and S. Grey lines are used to illustrate the segments that were isolated from the same virus strains. Hence, a similar topology for both segments should result in horizontal grey line. By contrast, oblique lines indicate dissimilarities of strain position, which most probably results from reassortments of viral segments between differentiated strains. Note that this pattern of dissimilarities seems to be much more frequent in the HNTV clade and the SEOV-GOUV-JURV clade than in the three other clades. ∗ indicate bootstrap values >80%.

Phylogenetic trees retrieved the major lineages currently described for *murinae*-associated hantaviruses group in the literature [46]. Each of them (except SAAV/DOBV) formed separate and well-supported phylogenetic lineages (PP > 0.95) (Figure 1). 

The phylogenies of segments M and S were generally congruent. The calculated Congruence Index (*Icong*, assessing the existence of topological congruence between the two trees [47,48]) is 2.99 (*p*-value = 1.2e−21), which indicates that the two trees were significantly more congruent than expected by chance. However, the Shimodaira-Hasegawa (SH) test [47,48] rejected the alternative topology (topology obtained with the other segment) for both segments (Δln L = 3824, *p* < 0.001 for S segment; Δln L = 12,444, *p* < 0.001 for M segment), indicating some incongruence between the trees, which were symbolized by oblique grey lines in Figure 1. Most incongruencies were observed within the HTNV and the SEOV-GOUV-JURV lineages. 

### 2.2. Molecular Signatures of Selection

In our study, we used five different models, the Single Likelihood Ancestor Counting (SLAC) model, the Fixed Effect Likelihood (FEL) model, the internal branches FEL (iFEL), the Mixed Effects Model of Evolution (MEME) and the Fast Unbiased Bayesian AppRoximation (FUBAR) to detect selection acting on both segments. Based on the methodology proposed in Wlasiuk and Nachman (2010) [49] and recommended by other authors [50], we chose to only consider sites that are identified by at least three of these methods as being under positive selection.

For the S segment, we found strong evidence of positive selection for only one aminoacid (aa) site in position 43 (Ala for DOBV, SEOV and HTNV) supported by the five methods (Table 1). However, the results of the MEME method results suggested that a larger number of sites may be subjected to episodic diversifying selection as it identified 21 others aa sites at 0.05 significance level. Codon analyses also revealed between 85% (SLAC, Single Likelihood Ancestor Counting model), and 98% (FUBAR, Fast Unbiased Bayesian AppRoximation model) of negatively selected aa sites, (*p* = 0.05 or Bayes Factor > 0.9) (Table 1). Results were consistent for both the small and the large dataset but 10 supplementary aa sites were detected by the MEME method with the large dataset (Table 1, see Experimental Section for description of the datasets).

**Table 1:** Signatures of selection in S and M segments detected by SLAC, FEL, iFEL, FUBAR and MEME methods. * indicate sites identified as being under positive selection by at least two methods.

Methods	S segment (small dataset)		S segment (large dataset)		M segment	
	positively selected aa sites	percentage of negatively selected aa sites	positively selected aa sites	percentage of negatively selected aa sites	positively selected aa sites	percentage of negatively selected aa sites
SLAC	43 *	85%	43 *	90%	no site	86%
FEL	43 *	89%	no site	92%	no site	90%
iFEL	43 *	88%	no site	91%	no site	90%
FUBAR	43 *	98%	43 *	98%	no site	97%
MEME	3, 4, 23, 39, 43 *, 69, 70, 71, 129, 183, 190, 209, 221, 233, 265, 313, 332, 334, 336, 357, 365, 369	not tested	2, 3, 4, 7, 23, 27, 39, 43 *, 69, 70, 75, 181, 209, 214, 221, 265, 310, 311, 312, 313, 326, 334, 336, 343, 365, 369, 396, 397, 398, 403, 405, 408	not tested	22, 29, 99, 189, 212, 208, 318, 340, 345, 377, 420, 461, 462, 540, 544, 635, 642, 643, 725, 766, 780, 810, 817, 821, 877, 942, 969, 995, 1003, 1048	not tested

For the M segment, we were unable to obtain evidence of positive selection for some sites by at least three of the five methods. Still, the MEME method identified 30 aa sites as being potentially subjected to episodic diversifying selection events (Table 1). Except for seven of them (aa 461, 462, 540, 544, 635, 642 and 643) they all corresponded to aa located in the predicted ectodomains of the Gn and Gc glycoproteins [51,52,53]. As for the S segment, analyses revealed that the M segment was subjected to a strong purifying selection with between 86% (SLAC) and 97% (FUBAR) negatively selected aa sites (Table 1). 

### 2.3. Shifts in Diversification Rates and Patterns

For both M and S segments, our analyses revealed that diversification rates significantly varied along time. 

**Figure 2:**
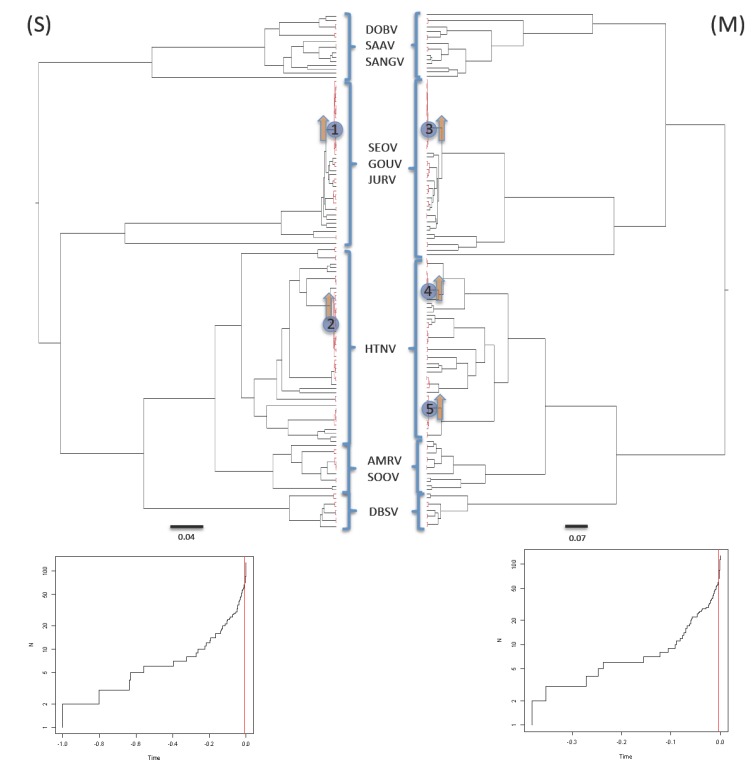
M and S segments of murine-associated hantaviruses BEAST time calibrated (ultrametric) trees. Root age was arbitrarily assigned to 1. A Generalized mixed Yule coalescent (GMYC) model was used to determine thresholds such that nodes before the threshold are considered as species diversification events, whereas branches crossing the thresholds define clusters following a coalescent process (in red). Shifts in diversification rates determined using MEDUSA algorithm are represented by numbered blue circles. Increases in lineage diversification rates among the tree compared to the reminder tree determined by the PRC Parametric Rate Comparison) test implemented in the iterates package are represented by orange arrows. Inserts illustrate the Lineage-through-time (LTT) plots based on the BEAST time calibrated (ultrametric) trees for the M and S segment of murine-associated hantaviruses in which the occurrence and the position of the switch between two evolution patterns (red line) was determined by the method of Pons *et al.* implemented in the package SPLITS [54].

The general mixed Yule coalescent (GMYC) model was preferred over the null model of uniform branching rates (2ΔL = 34.28, χ2 test *p* < 0.0001 and 2ΔL = 31.86, χ2 test *p* < 0.0001 for S and M segments respectively with the single-threshold analysis while the multiple-threshold analysis gave a result of 2ΔL = 37.59, χ2 test *p* < 0.0001 and 2ΔL = 35.62, χ2 test *p* < 0.0001 for S and M segments, respectively. This meant that the best model of branching patterns for both segments corresponded to a temporal succession of speciation processes followed by coalescent processes. This pattern is well illustrated by the lineage-through-time (LTT) plots, which evidence a sudden increase in branching rate towards the present (Figure 2). 

The positions of the switches in diversification rates were very close to the tip of the trees. Single- and multiple-threshold models gave essentially similar results (Supplementary Figure S1). The analyses lead to the identifications of many lineages affected by an increase in branching rates. The number of lineages identified by the method was very similar for both S and M segments; 60 putative lineages (confidence interval 53–67) and 66 putative lineages (confidence interval 54–81) respectively with the single-threshold analysis and 58 putative lineages (confidence interval 57–58) and 58 putative lineages (confidence interval 23–60), respectively, with the multiple-threshold analysis. Analyses performed with the large dataset indicated estimates of 32 lineages (confidence interval 21–113) with the single threshold method (2ΔL = 11.65, χ2 test *p* < 0.01) and 102 lineages (confidence interval 98–102) with the multiple threshold method (2ΔL = 20.86, χ2 test *p* < 0.0001). Finally, the number and composition of putative lineages identified by the PTP (Poisson Tree Processes) test were very similar to results obtained with the GMYC method for both S and M segments (60 and 65 putative lineages, respectively). This suggests that only minor biases caused by the ultrametrization process with BEAST occurred. 

The MEDUSA method allowed identifying where diversification rate shifts occurred on the tree. The analyses revealed two significant shifts for both M and S segment phylogenies. The location of the shifts was highly congruent between the two segments (Figure 2). One shift occurred in the clade including the SEOV strains from the Hubei Chinese province (shift 1 in segment S and 3 in segment M), and the second one encompassed the HTNV strains from the Guizhou Chinese province (shift 2 in segment S and 4+5 in segment M). In addition to these two clades, the analyses of the large dataset detected another shift occurring earlier in the phylogeny and affecting a large clade including SEOV strains from Asia (China, Vietnam and Singapore) and Europe (France and Belgium). The PRC method confirmed that the SEOV-Hubei clade and the HTNV-Guizhou clade have diversified more rapidly than lineages in the rest of the tree. 

### 2.4. Geographic Structuration Analyses

**Figure 3:**
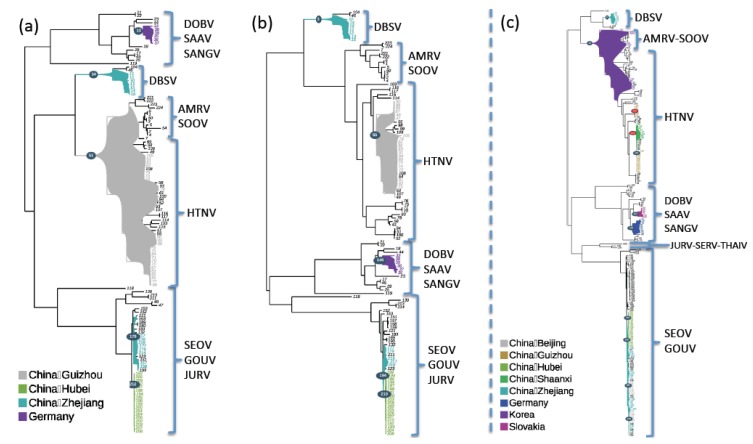
Detection of phylotypes (subsets of taxa with close phylogenetic relationships and common geographic trait values) among the phylogenies of M (**a**) and S, for the small dataset (**b**) and for the large dataset (**c**), segments of murine-associated hantaviruses by Phylotype [55]. Results are presented on the ML phylogenetic trees, in background and in which the selected phylotypes are represented by coloured regions. Phylotype identifiers are indicated in circles and refer to Pi in Table S2.

Parsimony reconstructions as implemented in PhyloType, yielded nearly identical results for both S and M segments phylogenies independently of the optimization method used (ACCTRAN or DELTRAN) (Figure 3, Table S2). 

Five phylotypes were identified in the reconstructions. Two phylotypes were recovered among the SEOV strains lineages, respectively, associated with Hubei (China) [Pi 210 (S) or 212 (M)] and Zhejiang (China) [Pi 194 (S) or 179 (M)] annotations. Another phylotype including all DBSV virus strains from Zhejiang was retrieved [Pi 3 (S) or 34 (M)], illustrating the geographical clustering of DBSV shown in [28]. Among the HTNV strains, a phylotype associated with the Guizhou annotation in the S phylogeny (Pi 55) was found at a more basal position on the M phylogeny (Pi 51), including a common ancestor of HTNV and AMRV/SOOV strains. The last phylotype was associated with the Germany annotation [Pi 146 (S) or 13 (M)] in the DOBV clade.

These phylotypes were also observed when using the larger dataset for the S segment, which allowed reaching the minimal size selection criteria for the detection of several additional phylotypes on the whole tree. In particular, a phylotype corresponding to the Korea annotation (Pi 22) including the common ancestor of AMRV/SOOV and HTNV strains, suggested a Korean ancestor for the considered HTNV strains, which is consistent with the known history of the HTNV discovery in 1976 in Korea [56]. 

The use of the large dataset also allowed obtaining two additional phylotypes among the SEOV clade associated with the Beijing annotations (Pi 393 and 423) and inferring the origin of the Zhejiang phylotype (Pi 318) earlier in the phylogeny, at an ancestral position compared to Hubei (Pi 319) and Beijing phylotypes.

Analyses also showed strong associations (*p* < 0.05) between diversification rate shifts and geographical origins in the S and M segments phylogenies. Guizhou and Hubei phylotypes (Pi 151 and 319, respectively, on Figure 4c and Table S2) correspond respectively to HTNV and SEOV lineages affected by diversification rate shifts as identified by MEDUSA. Moreover, the PhyloType results suggested that for the Hubei phylotype, the diversification rate shift event was accompanied by geographic shift of the ancestral strain (from Zhejiang, China to Hubei, China).

## 3. Discussion

Our phylogenetic analyses revealed changes in patterns of diversification and selection during the evolutionary history of RNA viruses. Our analyses suggest that RNA virus molecular variation is under strong evolutionary constraints, as approximately 90% of aa sites in both M and S segments are under negative selection. However, we also detected few aa sites belonging to the envelope glycoproteins and to the nucleoprotein under positive selection, putatively as a result of selection pressures exerted by hosts’ immunity. Moreover, our findings suggest that Hantavirus lineages mostly evolve under a birth and death process, as suggested in the literature for RNA viruses [57]. However, we also detected some acceleration of viral diversification in certain geographic areas. This change in diversification rates and patterns could be related to environmental and climatic characteristics that may influence transmission, generation time and/or replication of viruses in their host population [35]. This could locally increase the evolutionary potential of hantaviruses and thus enhance the risk of host jumping by providing the genetic and phenotypic variation needed for a rapid adaptation to a new host species [35,57] and, finally, the risk of disease emergence in humans [58,59].

### 3.1. Molecular Signatures of Selection

Molecular selection is caused by the pressure induced by the environment (including hosts) on the viral variants [60]. As viral genome is very compact and codes for important biological functions, there is often remarkable sequence conservation, indicating that purifying selection is a dominant evolutionary force in viral evolution, preserving aa residues, while allowing neutral variation in nucleotide sequences to continue [61].

In accordance with previous studies [27,28,29], we show that murine-associated hantaviruses S and M segments are mainly subject to purifying selection along their evolutionary history. For both segments, we detected significant negative selection for 1344 or 1525 aa sites (depending on the method used) over a total of 1562 (*i.e*., 88%–97% of the S and M segment aa sites). Only one aa was significantly detected as evolving under positive selection on the S segment while none was detected on the M segment. However, the MEME method allowed us to detect several sites that could have been subjected to episodic diversifying selection (22 on the S segment and 30 on the M segment), *i.e*., occurring locally during hantavirus evolution and confined to a small subset of branches of the phylogenetic tree. These sites were not detected in previous works [27,28], but Murrell *et al.* [62] suggested that many previous estimates of the proportion of sites under positive selection should be revised as the new powerful method MEME indicates that selection acting at individual sites is considerably more widespread than suggested by older constant models. They also suggested that natural selection is predominantly episodic, with transient periods of adaptive evolution masked by the prevalence of purifying or neutral selection on other branches [62], which is in agreement with our findings. Hantavirus evolutionary processes are subject to local dynamics that occur on a short time scale and also follow very complex patterns, which might differ between the very local scale and the global scale. 

Interestingly, most of the corresponding aa detected on the M segment (23/30) are part of the predicted ectodomain of the Gn (mapped between aa 17 and 441) and Gc (mapped between aa 650 and 1097) glycoproteins [51,52,53] of hantaviruses, which forms projections on the virion surface and are the targets of neutralizing antibodies [63,64], whilst the aa 43 detected on the nucleoprotein is part of a region identified as an antibody epitope for Sin Nombre (or Four Corners virus) [65], Hantaan [66] and Puumala [67] virus. This is consistent with an adaptation to host immune recognition in the *N*-terminal immunodominant region of the nucleocapsid protein [27]. Diversifying selection at these aa sites are thus probably the result of adaptation of Hantavirus lineages to their host species and the evolutionary pressure exerted by their immune systems. However, there is still a lack of information concerning the exact structure and interaction map of hantaviruses proteins. Structural studies of the hantaviral N proteins have progressed slowly because of their strong tendency to form aggregates hampering the efforts to crystallize them [68]. More data, from crystallography studies, are needed in order to better estimate the selective pressures acting on aa sites and understand the reason why some sites were found to be under positive selection.

### 3.2. Co-Divergence between S and M Segments

Co-divergence between viral segments is expected if segments from the same viral strains remain associated though time. By contrast, reassortment (*i.e*., exchange of segments) and/or recombination between segments of different strains may blur the pattern and cause departure from co-divergence. Both require that cells be infected by different strains at the same time. Reassortment and/or recombination events have already been reported in the literature for hantaviruses [25] and other RNA viruses (for a review of these cases, see Han 2001 [69]). Time-series like those of Razzauti [26] on hantaviruses yet suggest that the reassortants do not persist over time, presumably because they underwent negative selection. 

We found clear signals of departure from co-divergence in some lineages like the HTNV lineage and the SEOV-GOUV-JURV lineage, while other lineages were less impacted. Most departures from co-divergence were observed in the shallow nodes of our trees, *i.e*., within species and within major lineages. Yet, we also noticed two events between highly differentiated lineages, in particular between HTNV and SEOV, as already discussed by Zou *et al.* [18]. Our results thus suggest that cell co-infection by different lineages and/or negative selection against reassortants and/or recombined strains may vary across lineages and/or geographic regions. This observation deserves further studies because it could be related to different evolutionary dynamics and to possible risks of emergence in humans.

### 3.3. Shifts in Diversification Rates

Previous studies suggested that high rates of lineage births and deaths are a dominant feature of RNA virus evolution [57,70]. Our findings fully support this view, as the BD model is preferred to other models when considering the murine-associated hantaviruses phylogeny as a whole. However, we also detected several shifts to the Yule (pure-birth) model in certain tips of the phylogenetic trees. These shifts were detected in both S and M segments phylogenies and their localisations in the trees were congruent for both segments.

In particular, two shifts were identified with both S (small and large dataset) and M segments, both shifts corresponded to a change of the diversification process from a BD model to a Yule model. These changes were accompanied by a strong increase in diversification rates. One shift concerns the SEOV strains from the Hubei Chinese province and the second corresponds to a cluster including HTNV strains from the Guizhou Chinese province. Both clades have diversified more rapidly than the rest of the tree. Moreover, the two clades are geographically restricted (see also further) illustrating that in Hantavirus, rates and patterns of diversification may vary locally as already described for rabies virus [35] or tick-borne encephalitis virus [71]. As for these other RNA viruses, ecological and environmental conditions (such as climate, host dynamics and host jumping) could trigger the speed of viral diversification and its evolutionary dynamics.

These findings highlight the complexity of the diversification processes associated with the evolution of hantaviruses at different spatial and time scales and underline the contrast existing between micro- and macroevolution in RNA viruses. Generally, microevolution refers to rapid changes within populations over time, while macroevolution refers to long-term changes driving the evolution of new species [72]. Our results are in agreement with the findings by several authors that long-term viral substitution rates (rates in deep nodes of the phylogeny) can be orders of magnitude lower than short-term substitution rates (rate estimates at the tips of phylogenies) [61]. Strong purifying selection (as we observed for murine-associated hantaviruses) could be responsible for this discrepancy. As a result, this could also translate into an underestimation of branch lengths in the deepest branches of the phylogenies, if we calibrate the tree with substitution rates inferred from the tip of the trees.

### 3.4. Ecological Drivers of the Diversification Rate Variability

Determinants of evolutionary rate variability among closely related viruses or among lineages of the same viral species circulating in different geographic regions or host species are still poorly understood. Changes in host dynamics, geographic dispersal events or jumps between host species may enhance viral diversification because of genetic bottlenecks and/or changes in selective pressures exerted by the host immune systems [19,33,73]. As a result, a virus shifting between host species (or lineages) usually evolves faster than a virus confined to a single host, because viral replication within a host over time decreases under the pressure of the host immune system [74]. Viral diversity then decreases as a result of genetic bottleneck or genetic adaptation to the host immune systems. For the same reasons, viral diversification is expected to be lowered during chronic infection following the acute phase of infection [75], a widely held assumption for hantavirus infection in rodents [76]. New environmental conditions can also shift the equilibrium between viral and host population and favour rapid viral diversification [77,78]. In the rabies virus, the seasonality of transmission in different climatic regions strongly influences viral diversification, probably via climate-related changes in host dynamics [35]. Similarly, variation of local precipitation, humidity and temperatures has been correlated to HFRS incidence in Shandong Chinese province [79] probably by influencing hantavirus transmission among their rodent hosts [80]. 

From this point of view, Hubei and Guizhou provinces, where we observed high diversification rates for SEOV and HNTV, present interesting geographic and climatic characteristics [81]. Hubei is a province located in central south China, where the main host of SEOV, the brown rat, *Rattus norvegicus*, probably originated (given fossil records, Wu *et al.* 2008 cited in [82]). Rat communities there are characterised by the coexistence of many highly divergent genetic lineages [82]. Recent studies showed that this high genetic diversity in rats is paralleled by a high genetic diversity in SEOV [82]. Finally, Hubei province is crossed by the Yangtze river, which is an important trade route for humans, favouring rat dispersal and high mixing of both Hantavirus and rat lineages [83,84]. Based on above knowledge gained from other RNA viruses, we could thus hypothesise that such mixing of rodent and Hantavirus lineages could accelerate hantavirus diversification, but this deserves further studies. One can notice that Hubei province is one of the most severe epidemic areas of SEOV-related Haemorrhagic Fever with Renal Syndrome (HFRS) for humans in China.

Guizhou province, located in southern part of China, is another major endemic area for HTNV-related HFRS in humans, and HTNV hantaviruses display there a high degree of genetic diversity [18,81]. In Guizhou province, as well as in Hubei province, recent works have documented frequent spillover events of HTNV and SEOV between *Apodemus agrarius*, *Rattus niviventer* and *Rattus norvegicus* [18,82]. Natural reassortments between HTNV and SEOV have also been documented [18]. Cross-species transmission seems to be an important driver of Murinae-associated hantaviruses evolution [28]. The geographic localization as well as environmental and/or climatic conditions favouring intense circulation and contacts between different potential host species and Hantavirus lineages could therefore be at the origin of the increase of diversification rates we observed there for HTNV and SEOV. 

### 3.5. Sampling Effects

In this work, we chose to use only strains for which relevant genetic data (complete segment sequences) were available. This probably results in strong geographic and lineage biases, because not all geographic regions and genetic lineages have been studied equally. For example, strains from some Chinese provinces or from other Asian or European countries are underrepresented in our dataset compared to Guizhou and Hubei provinces. Such bias could result in underestimation of the number and localisation on clades experiencing drastic changes in patterns of diversification [85]. Indeed, we detected an additional clade experiencing an increase in diversification rate when we used the large S dataset. Moreover, the number of geographic phylotypes detected is much more important with the large S dataset, probably because a minimal number of strains is needed to be able to extract significant information when using the method of Chevenet *et al.* [55]. Another limitation of our study is that our analyses are dependent on the metadata linked with each deposited sequence, such as the precise time and place of sampling (latitude and longitude) that are often missing or incomplete in GenBank [30]. Indeed, more precise geographic data could help to better refine the geographic areas where changes in evolutionary dynamics occur. Nevertheless, despite these obvious limitations we provide here a strong indication of the existence of different evolutionary patterns across lineages. For instance, though a large dataset was available for the DOBV/SAAV clade (29 sequences available in the large dataset *vs.* 15 in the small dataset), we were not able to detect any change in diversifying rates. Such a result contrasts with what was observed for HTNV and SEOV clades in China. 

## 4. Experimental

### 4.1. Sequences Acquisition

We used a dataset of 124 Hantavirus strains belonging to the hantaviruses carried by the Rodentia subfamily Murinae. We selected only strains for which complete coding sequences for the two segments (S, ORF, 1293 bp, and M, ORF, 3408 bp) were available. Segment L was not included in the analyses due to the paucity of the data available (8 SEOV, 1 SANGV, 9 HTNV and 11 DOBV). For some analyses we also used a larger dataset of 225 complete S segments for which the M segment was not available. Sequences were obtained from the NIAID Virus Pathogen Database and Analysis Resource (ViPR) online through the website in [86]. The list of strains and their geographic origin are displayed in Table S1 and Figure S1. 

### 4.2. Molecular Signature of Selection

To detect and/or determine selection acting on both segments, sequences were analyzed using HyPhy software package [87,88,89] implemented in the Datamonkey web server [90]. Datamonkey includes several codon-based maximum-likelihood methods described in [87,88], inferring both positively and negatively selected sites: the Single Likelihood Ancestor Counting (SLAC) model, the Fixed Effect Likelihood (FEL) model, the internal branches FEL (iFEL). SLAC is the most conservative of the three methods. We also employed two recently developed models, the Mixed Effects Model of Evolution (MEME) [62] and the Fast Unbiased Bayesian AppRoximation (FUBAR) [91] methods. MEME is a branch-site model, which is an extension of FEL that permits a proportion of branches at a site to evolve neutrally or under negative selection, while the remainder can evolve under diversifying selection. It can therefore detect signatures of episodic selection even when the majority of lineages are subject to purifying selection. FUBAR is a very fast approximate hierarchical Bayesian method using a Markov chain Monte Carlo (MCMC) routine that ensures robustness against model misspecification by averaging over a large number of predefined site classes. 

Significance levels were set fixed to 0.05 or 0.9 (Bayes factor) and the REV substitution model (also called General Time Reversible model) with a Neighbour Joining tree as default tree.

### 4.3. Phylogeny and Congruence between M and S Segment Trees

Sequences were aligned with the Clustal Omega alignment program implemented in SEAVIEW v4.4.2 [92]. The optimal substitution model for both segments was identified as the GTR +G +*I* model (General Time Reversible) using a function implemented in Mega v5.1 [93]. Phylogenetic reconstructions were conducted with the ML approach using PhyML v3.0 [94]. We tested the congruence between the S segment phylogeny and the M segment phylogeny using the on-line calculation of the congruency index *Icong* [47] see [95]. This index is based on topological congruence assessed using MAST (Maximum agreement subtrees) measurement. A large number of random trees are generated, with a wide range of number of leaves and the topological congruence is then determined as the minimum number of leaves that have to be removed in each phylogeny to render the trees identical. A *p*-value is given for the null hypothesis that two given trees are not more similar than expected by chance. We also conducted comparative tests of topologies by ML using the Shimodaira-Hasegawa (SH) test implemented in PAUP* [96] and using 1000 RELL (Resampling estimated log-likelihoods) bootstraps [48]. Tanglegram in which S and M segment phylogenetic trees are drawn opposite each other with lines connecting matching taxa, was visualized using TreeMap v.3 [97]. 

### 4.4. Phylogenetic Analyses

Diversification analyses require the use of ultrametric trees as input trees. Ultrametric trees for both segments were obtained using the Metropolis-coupled Markov Chain Monte Carlo (MCMC) method in BEAST package v1.7.4 [98]. BEAUTi v1.7.4 [98] was used to generate BEAST .xml input files. The two datasets were analysed under the GTR +G +*I* model and with a relaxed clock allowing branch lengths to vary according to an uncorrelated lognormal distribution. Inferring ultrametric trees with BEAST for species-level phylogenies requires specifying a speciation model prior [99]. Two most used speciation models are available in BEAST, the pure-birth (Yule) process, which governs branching times assuming a constant rate of diversification and no extinction [100]; and the birth-death (BD) process, which is a special case of Yule process allowing lineage extinction. In order to search for the model that best fits to our data sets, we used the *fitdAICrc* function (which compares models AIC scores) implemented in the R package Laser [101] on the S and M ML phylogenetic trees previously made ultrametric by the *chronos* function implemented in the R package Ape [102]. As the birth-death (BD) process was selected for both segments S and M (Table 2), we then used a BD prior for ultrametric tree inference with BEAST, and all other priors were left to the default settings. 

**Table 2:** Likelihoods, and AIC scores calculated by the *fitdAICrc* function implemented in the « Laser » R package for both Yule and Birth-death models. Models are fitted to the M and S segment phylogenetic trees constructed with PhyML and made ultrametric by the *chronos* function implemented in the « Ape » R package.

Model	Segment S		Segment M	
	LH	AIC	LH	AIC
Pure birth (Yule)	537.98	−1073.96	674.65	−1347.31
Birth death (BD)	540.18	−1076.36	755.33	−1506.7

A random tree was used as the starting tree. Prior ages to lineages were arbitrarily assigned to 1 (meaning that the time separating the root from the present was arbitrarily assigned to 1). We ran two analyses of 10 million generations each for both segments with parameters sampled every 1000 generations. Convergence was evaluated using Tracer v1.5 [103] and summary maximum clade credibility trees (MCC) were generated using TREEANNOTATOR v1.7.4 [98], after discarding the first 10% of the trees in each run as burn-in as determined graphically using Tracer v1.5.

### 4.5. Shifts in Diversification Rates and Patterns

We ran several analyses to identify shifts in modes of lineage diversification in both S and M phylogenies. Analyses were performed in R (R-Development-Core-Team 2008) using the MCC trees and the Ape [102], Geiger [104], Laser [101], SPLITS [54] and iteRates [105] libraries. 

The Species’ LImits by Threshold Statistics model of Pons *et al.*, 2006 [54] implemented in the package SPLITS seeks for transition between two branching patterns in a tree, corresponding to the Yule process and the coalescent process, respectively. The method tests for the fit of the data to the null model of general coalescence (in which the entire tree represents a single population) and to the alternative model, called general mixed Yule coalescent (GMYC) model, which combines models that separately describe branching within populations (coalescent process) and branching between species (a Yule model of stochastic lineage growth). A standard likelihood ratio test (LRT) is used to assess whether the alternative model provides a better fit to the data than the null model. However, as a single threshold approach may not reflect the variety of divergence levels among lineages, we also applied a multiple threshold approach that allows the depth of the coalescent transition to vary along individual branches of the phylogenetic tree [106].

We also used the Poisson Tree Processes (PTP) model for delimiting species on a rooted phylogenetic tree [107]. In PTP, speciation or branching events are modelled in terms of number of substitutions (represented by branch lengths), so it only requires a phylogenetic input tree. We used PTP test to assess whether the ultrametrization process with BEAST could introduce bias in our analyses, by inferring putative species boundaries on both S and M segment phylogenetic trees. Analyses were conducted on the web server for PTP [108] using phylogenetic trees obtained with RAxML v7.0.3 [109] under a GTR +CAT +I substitution model. The latter model is an alternative of the +G model of rate heterogeneity, which optimizes the evolutionary rate for each individual site using a fixed number of rate categories. 

The Modelling Evolutionary Diversification Using Stepwise AIC (MEDUSA) method implemented in the package GEIGER aims to detect and locate lineage-specific shifts in diversification rates in a tree. To this aim, it fits a series of birth-death (BD) and pure-birth Yule models to a phylogenetic tree (reconstructed by default under a BD process) with an increasing number of breakpoints (rate shifts) [110]. These models are then compared using corrected Akaike information criterion (AICc) to choose the best-fit model. We tested for significant variation in diversification rates by using the medusaSummary command and chose the model with the lowest AICc value. The threshold for retaining additional rate shifts was an improvement in AIC score of 5.26 units or greater as determined as appropriate for a tree of 124 tips by MEDUSA. We also used the Parametric Rate Comparison (PRC) test implemented in the “iterates” package [105]. The method is similar to the MEDUSA approach but does not make assumptions regarding the underlying evolutionary processes of diversification.

### 4.6. Geographic Structuration Analyses

Finally, we aimed to relate the shifts in diversification rates in our phylogenetic trees with the geographic distribution of the lineages. To this aim we used the PhyloType software [111]. The method allows detecting genetic clusters, thereafter called *phylotypes*, which include strains that share similar extrinsic traits (e.g., geographic location, morphology, host species or the presence of a given mutation, *etc*.) [55]. Phylotypes are thus subsets of strains with close phylogenetic relationships and common trait values. The method combines ancestral trait reconstruction using parsimony, with combinatorial and numerical criteria measuring tree shape characteristics and the diversity and separation of the potential phylotypes. A shuffling procedure is used to assess the statistical significance of phylotypes. 

Analyses were performed with phylogenies inferred by maximum likelihood (ML) and with a LRT SH-like branch support as described in [55]. Annotations in our dataset were the geographic origin of each strain. For all analyses, criteria chosen for phylotype selection were as follows: size ≥5, size/different ≥1, persistence ≥1, only nodes which ML bootstrap values were ≥75 were taken into account in the analyses. Both ACCTRAN and DELTRAN (recommended for geographic annotations) optimization were tested. Shuffling procedures were performed with 1000 iterations to test for phylotype significance. Only those phylotypes whose *p*-value was ≤5% were retained. 

## 5.Conclusions

The diversification of Murinae-associated hantaviruses follows a very complex pattern which might differ at the very local scale and at the global scale. As RNA viruses’ evolutionary processes are subject to local ecological dynamics that occur on a short time scale, a credible model of virus evolution has to take time-dependent ecological processes into account [112]. As already pointed out by Holmes 10 years ago [70], there is still a great need for more sophisticated models of RNA virus sequence evolution that allow patterns and rates to differ among lineages [70]. Within some areas, like Hubei and Guizhou province in China, geographic and climatic characteristics allow intense cocirculation of hosts and Hantavirus lineages, favouring the occurrence of spillover, and accelerating hantavirus diversification rates and genetic novelty. These conditions are probably suitable for the emergence of new genetic variants and increase the risk of transmission to humans. Indeed, those two provinces of China are important foci of human hantaviruses. One should notice that hantavirus variation is just about to be described in many other places in the world and most Hantavirus species are poorly or not documented, preventing similar analyses in other areas and with other species. Such studies should be strengthened in the future aiming to better predict the geographic or origins of epidemics. 

## Supplementary Files

Supplementary File 1 Supplementary Materials (PDF, 1415 KB)
