# Supplementary material for: Changes in Diversification Patterns and Signatures of Selection during the Evolution of Murinae-Associated Hantaviruses
Source: Viruses. 2014 Mar 10;6(3):1112–34. doi: 10.3390/v6031112 (PMC3970142; doi:10.3390/v6031112)
Supplement: Supplementary File 1 — Supplementary Materials (PDF, 1415 KB) [file viruses-06-01112-s001.pdf]

Supplementary Materials for

Changes in Diversification Patterns and Signatures of Selection during the Evolution of Murinae-Associated Hantaviruses

Table S1. Hantavirus strains used for analysis in this study.

Species	Strain	Localisation	Ref Seg S	Ref seg M	Code
Amur	ApJLCB2011-99	China-Jilin	JX473004	JX473003	1
Amur	B78	China-Shandong	AB127997	AB127994	2
Amur	H5	China-Heilongjiang	AB127996	AB127993	3
Amur	H8205	China-Heilongjiang	KC136244	KC136243	4
Amur	JilinAp06	China-Jilin	EF121324	DQ914791	5
Amur	JLCB2011	China-Jilin	JX119010	JX119009	6
Amur	Khekhtsir/AP209/2005	Russia-Khabarovsk	AB620028	AB620029	7
Amur	NA33	China-Heilongjiang	JQ061291		8
Amur	Solovey/AP63/1999	Rusia-Primorye	AB071184		9
Dabieshan	Wencheng-Nc-427	China-Zhejiang	JF796017	JF796031	10
Dabieshan	Wencheng-Nc-469	China-Zhejiang	JF796018	JF796032	11
Dabieshan	Wencheng-Nc-470	China-Zhejiang	JF796019	JF796033	12
Dabieshan	Yongjia-Nc-15	China-Zhejiang	JF796020	JF796034	13
Dabieshan	Yongjia-Nc-38	China-Zhejiang	JF796021	JF796035	14
Dabieshan	Yongjia-Nc-58	China-Zhejiang	JF796022	JF796036	15
Dabieshan	Yongjia-Nc-95	China-Zhejiang	JF796023	JF796037	16
DOBV	3970/87	Slovenia	L41916	L33685	17
DOBV	Aa1854	Russia - Lipetsk	EU188452	EU188453	18
DOBV	Ap/Sochi/43	Russia - Sochi	JF920151		19
DOBV	Ap/Sochi/79	Russia - Sochi	JF920152		20
DOBV	Ap/Sochi/hu	Russia - Sochi	JF920150	JF920149	21
DOBV	AP1584	Russia - Sochi	EU188449	EU188450	22
DOBV	DOB/Saaremaa/160V	Estonia	AJ009773	AJ009774	23
DOBV	DOBV/Ano-Poroia/13Af/99	Greece	AJ410619		24
DOBV	DOBV/Ano-Poroia/Afl9/1999	Greece	NC005233	AJ410616	25
DOBV	East Slovakia-856-Aa	Slovakia	AJ269549		26
DOBV	East Slovakia-862-Aa	Slovakia	AJ269550		27
DOBV	East Slovakia/400Af/98	Slovakia	AY168576	AY168577	28
DOBV	Esl/29Aa/01	Slovakia	AY533118		29
DOBV	Esl/34Aa/01	Slovakia	AY961618		30
DOBV	Esl/81Aa/01	Slovakia	AY533120		31
DOBV	GER/05/239/Aa	Germany	GQ205405		32
DOBV	GER/05/477/Af	Germany	GQ205406	GQ205411	33
DOBV	GER/07/1064/Aa	Germany	GQ205404		34
DOBV	GER/07/293/Aa	Germany	GQ205401	GQ205409	35
DOBV	GER/07/607/Af	Germany	GQ205402	GQ205410	36
DOBV	GER/08/118/Aa	Germany	GQ205407	GQ205412	37
DOBV	GER/08/131/Af	Germany	GQ205408	GQ205413	38
DOBV	GRW/Aa	Germany	JQ026204	JQ026205	39
DOBV	Kurkino/44Aa/98	Russia - Kurkino	AJ131672		40
DOBV	Kurkino/53Aa/98	Russia - Kurkino	AJ131673		41
DOBV	Saar/90Aa/97	Estonia	AJ009775		42
DOBV	Saaremaa/Lolland/Aa1403/2000	Denmark	AJ616854		43

Table S1. *Cont.*

Species	Strain	Localisation	Ref Seg S	Ref seg M	Code
DOBV	SK/Aa	Slovakia	AY961615	AY961616	44
DOBV	Slo/Af-BER	Slovenia	GU904029	GU904035	45
Hantavirus	AH09	China-Anhui	AF285264	AF285265	46
Hantavirus	CGRn8316	China-Guizhou	EF990903	EF990917	47
Hantavirus	CGRn9415	China-Guizhou	EF990902	EF990916	48
hantavirus	KY	China-Yunnan	GU140098	GU140097	49
Hantavirus	Liu	China-Shandong	AF288649	AF288648	50
Hantavirus	S85-46	China-Shichuan	AF288659	AF288658	51
HTNV	76-118	SouthKorea	M14626	M14627	52
HTNV	84FLi	China-Shaanxi	AY017064	AF345636	53
HTNV	A16	China-Shaanxi	AF288646	AF288645	54
HTNV	A9	China-Jiangsu	AF329390	AF035831	55
HTNV	AA1028	Russia-Khabarovsk	AF427318		56
HTNV	AA2499	Russia-Khabarovsk	AF427320		57
HTNV	AA57	Russia-Khabarovsk	AB620031	AB620032	58
HTNV	AP1371	Russia-Khabarovsk	AF427324		59
HTNV	AP708	Russia-Khabarovsk	AF427322		60
HTNV	Bao14	China-Heilongjiang	AB127998	AB127995	61
HTNV	CA09082007	China-Shaanxi	HQ834499		62
HTNV	CA10081109	China-Shaanxi	HQ834500		63
HTNV	CA10081113	China-Shaanxi	HQ834501		64
HTNV	CA10081203	China-Shaanxi	HQ834502		65
HTNV	CA10081206	China-Shaanxi	HQ834503		66
HTNV	CA10081708	China-Shaanxi	HQ834504		67
HTNV	CA10081905	China-Shaanxi	HQ834505		68
HTNV	CFC94-2	Korea	X95077		69
HTNV	CGAa1011	China-Guizhou	EF990913	EF990927	70
HTNV	CGAa1015	China-Guizhou	EF990912	EF990926	71
HTNV	CGAa2	China-Guizhou	EU092219	EU092223	72
HTNV	CGAa31MP7	China-Guizhou	EF990911	EF990925	73
HTNV	CGAa31P9	China-Guizhou	EF990910	EF990924	74
HTNV	CGAa4MP9	China-Guizhou	EF990915	EF990929	75
HTNV	CGAa4P15	China-Guizhou	EF990914	EF990928	76
HTNV	CGAa75	China-Guizhou	EU092220	EU092224	77
HTNV	CGHu1	China-Guizhou	EU092218	EU092222	78
HTNV	CGHu2	China-Guizhou	EU363813	EU363819	79
HTNV	CGHu3	China-Guizhou	EU363809	EU363818	80
HTNV	CGHu3612	China-Guizhou	EF990909	EF990923	81
HTNV	CGHu3614	China-Guizhou	EF990908	EF990922	82
HTNV	CGRn15	China-Guizhou	EU363810	EU363814	83
HTNV	CGRn2616	China-Guizhou	EU363811	EU363816	84
HTNV	CGRn2618	China-Guizhou	EU363808	EU363817	85
HTNV	CGRn45	China-Guizhou	EU092221	EU092225	86
HTNV	CGRn53	China-Guizhou	EF990907	EF990921	87
HTNV	CGRn5310	China-Guizhou	EF990906	EF990920	88
HTNV	CGRn93MP8	China-Guizhou	EF990905	EF990919	89

Table S1. *Cont.*

Species	Strain	Localisation	Ref Seg S	Ref seg M	Code
HTNV	CGRn93P8	China-Guizhou	EF990904	EF990918	90
HTNV	CGRni1	China-Guizhou	EU363812	EU363815	91
HTNV	Chen4	China-Shaanxi	AB027101		92
HTNV	CJAp93	China-Jilin	EF208929	EF208930	93
HTNV	CUMC-B11	SoutKorea	U37768	U37729	94
HTNV	E142	China-Yunan	AF288644		95
HTNV	Galkino/AA57/2002	Russia-Khabarovsk	AB620031		96
HTNV	H10150	China-Shaanxi	HQ834506		97
HTNV	HubeiHu02	China-Hubei	JQ665905	JQ665881	98
HTNV	HV004	China-Hubei	JQ083395	JQ083394	99
HTNV	LR1	China	AF288294	AF288293	100
HTNV	Maaji-1	Korea	AF321094		101
HTNV	Maaji-2	Korea	AF321095		102
HTNV	N8	China-Jiangxi	DQ658415	EF077656	103
HTNV	NC167	China-Anhui	AB027523	AB027115	104
HTNV	Q32	China-Guizhou	AB027097	DQ371905	105
HTNV	RG9	China-Guangdong	AF288296		106
HTNV	SN7	China-Shichuan	AF288657	AF288656	107
HTNV	TJJ16	China-Tianjin	AY839871	EU074672	108
HTNV	WuhanAaJ10	China-Hubei	JQ665906	JQ665882	109
HTNV	YaluRiver13	China-Jilin	HQ611981		110
HTNV	YN509	China-Yunnan	GU329991		111
HTNV	YU61	China	AY748308		112
HTNV	YU62	China	AY748309		113
HTNV	Z10	China-Zhejiang	AF184987	AF143675	114
HTNV	Z251	China-Zhejiang	EF595840	GQ120966	115
HTNV	ZLS-12	China-Zhejiang	FJ753398	FJ753399	116
HTNV	ZLS6-11	China-Zhejiang	FJ753396	FJ753397	117
Jurong	TJK/06	Singapore	GQ274941	GQ274939	118
SANGV	SA14	Guinea	JQ082300	JQ082301	119
SANGV	SA22	Guinea	JQ082303		120
SEOV	80-39	Sout Korea	S47716	NC005237	121
SEOV	93HBX12	China-Hebei	EF192308		122
SEOV	BjHD01	China-Beijing	AY627049	DQ133505	123
SEOV	Cherwell	UK	KC626089		124
SEOV	CixiRf23	China-Zhejiang	FJ803201		125
SEOV	CixiRf56	China-Zhejiang	FJ803202		126
SEOV	CixiRn169	China-Zhejiang	FJ803207		127
SEOV	CixiRn21	China-Zhejiang	FJ803205		128
SEOV	CixiRn76	China-Zhejiang	FJ803206		129
SEOV	CSG5	Vietnam	AB618112		130
SEOV	CUI	China-Beijing	GQ279395		131
SEOV	DPRK08	North Korea	JX853575	JX853576	132
SEOV	FeixianRn1	China-Shandong	GU592942		133
SEOV	GanyuMm187	China-Jiangsu	GU592934		134
SEOV	GanyuRn137	China-Jiangsu	GU592933		135

Table S1. *Cont.*

Species	Strain	Localisation	Ref Seg S	Ref seg M	Code
SEOV	GanyuRn66	China-Jiangsu	GU592932		136
SEOV	GaomiRn47	China-Shandong	GU592938		137
SEOV	GaomiRn9	China-Shandong	GU592941		138
SEOV	Gou3	China-Zhejiang	AB027522	AB027521	139
SEOV	GuangzhouRn36	China-Guangdong	GU592948		140
SEOV	Hb8610	China-Shanxi	AF288643		141
SEOV	HebeiMm7	China-Hebei	GU592935		142
SEOV	HuBJ15	China-Beijing	GQ279390		143
SEOV	HuBJ16	China-Beijing	GQ279380		144
SEOV	HuBJ19	China-Beijing	GQ279389		145
SEOV	HuBJ20	China-Beijing	GQ279394		146
SEOV	HuBJ22	China-Beijing	GQ279379		147
SEOV	HuBJ3	China-Beijing	GQ279391		148
SEOV	HuBJ7	China-Beijing	GQ279381		149
SEOV	HuBJ9	China-Beijing	GQ279384		150
SEOV	HuludaoRn101	China-Liaoning	GU592952	GU592931	151
SEOV	Humber	UK	JX879769	JX879768	152
SEOV	IR461	UK	AF329388	AF458104	153
SEOV	JinanRn1	China-Shandong	GU592937		154
SEOV	JiningCt13	China-Shandong	GU592940		155
SEOV	JUN5-14	China-Shandong	DQ217791		156
SEOV	K24-e7	China-Zhejiang	AF288653	AF288652	157
SEOV	K24-v2	China-Zhejiang	AF288655	AF288654	158
SEOV	L99	China-Jiangxi	AF288299	AF288298	159
SEOV	Longwan581	China-Zhejiang	GU592946		160
SEOV	OuhaiRf35	China-Zhejiang	FJ803208		161
SEOV	OuhaiRn146	China-Zhejiang	FJ803210		162
SEOV	OuhaiRn189	China-Zhejiang	FJ803211		163
SEOV	OuhaiRn251	China-Zhejiang	FJ803212		164
SEOV	Pf26	China-Heilongjiang	AY006465		165
SEOV	QingdaoMm15	China-Shandong	GU592939		166
SEOV	QixianRn10	China-Henan	GU592949		167
SEOV	R22	China-Henan	AF288295		168
SEOV	REPLONGES/Hu/FRA/2012/12-0882	France	KC902522		169
SEOV	Rn-CP7	China-Beijing	GQ279382		170
SEOV	Rn-DC8	China-Beijing	GQ279386		171
SEOV	Rn-DH27	China-Beijing	GQ279393		172
SEOV	Rn-HD11	China-Beijing	GQ279392		173
SEOV	Rn-M11	China-Beijing	GQ279383		174
SEOV	Rn-SHY17	China-Beijing	GQ279388		175
SEOV	RuianRf74	China-Zhejiang	FJ803213		176
SEOV	RuianRn180	China-Zhejiang	GU592953	GU904035	177
SEOV	RuianRn23	China-Zhejiang	FJ803214		178
SEOV	RuianRn242	China-Zhejiang	GU592945	GU592928	179
SEOV	RuianRn33	China-Zhejiang	FJ803215		180
SEOV	RuianRn76	China-Zhejiang	FJ803216		181

Table S1. *Cont.*

Species	Strain	Localisation	Ref Seg S	Ref seg M	Code
SEOV	RuianRr57	China-Zhejiang	FJ803217		182
SEOV	Sapporo	Japan	M34881	M34882	183
SEOV	SC106	China-Heilongjiang	GU361893		184
SEOV	SD201	China-Shandong	GQ279385		185
SEOV	SEO/Belgium/Rn895/2005	Belgium	JQ898106		186
SEOV	ShenyangRn139	China-Liaoning	GU592951		187
SEOV	ShenyangRn19	China-Liaoning	GU592950		188
SEOV	Singapore/06(RN41)	Singapore	GQ274944	GQ274942	189
SEOV	Singapore/06(RN46)	Singapore	GQ274945	GQ274943	190
SEOV	tchoupitoulas	USA	AF329389		191
SEOV	WuhanMm13	China-Hubei	JQ665910	JQ665886	192
SEOV	WuhanMm24	China-Hubei	JQ665911	JQ665887	193
SEOV	WuhanRf02	China-Hubei	JQ665912	JQ665888	194
SEOV	WuhanRf07	China-Hubei	JQ665913	JQ665889	195
SEOV	WuhanRf08	China-Hubei	JQ665914	JQ665890	196
SEOv	WuhanRf11	China-Hubei	JQ665915	JQ665891	197
SEOV	WuhanRf12	China-Hubei	JQ665916	JQ665892	198
SEOV	WuhanRf18	China-Hubei	JQ665917	JQ665893	199
SEOV	WuhanRf33	China-Hubei	JQ665918	JQ665894	200
SEOV	WuhanRf49	China-Hubei	JQ665919	JQ665895	201
SEOV	WuhanRn10	China-Hubei	JQ665920	JQ665896	202
SEOV	WuhanRn25	China-Hubei	JQ665921	JQ665897	203
SEOV	WuhanRn53	China-Hubei	JQ665922	JQ665898	204
SEOV	WuhanRn57	China-Hubei	JQ665923	JQ665899	205
SEOV	WuhanRn58	China-Hubei	JQ665924	JQ665900	206
SEOV	WuhanRn63	China-Hubei	JQ665925	JQ665901	207
SEOV	WuhanRn67u	China-Hubei	JQ665926	JQ665902	208
SEOV	WuhanRn75	China-Hubei	JQ665927	JQ665903	209
SEOV	WuhanRn98	China-Hubei	JQ665928	JQ665904	210
SEOV	XiaotangshanRn7	China-Beijing	GU592944	GU592927	211
SEOV	YaluRiver12	North Korea	HQ611980		212
SEOV	YongjiaRf45	China-Zhejiang	GU592943	GU592926	213
SEOV	YongjiaRn14	China-Zhejiang	GU592947	GU592929	214
SEOV	YZG-Changchun	China-Jilin	EF536376		215
SEOV	Z37	China-Zhejiang	AY605933	AF190119	216
SEOV	ZT10	China-Zhejiang	AY766368	DQ159911	217
SEOV	ZT71	China-Zhejiang	AY750171	EF117248	218
SEOV	zy27	China-Heilongjiang	AF406965		219
Serang	Serang/Rt60/2000	Indonesia	AM998808		220
Soochong	SC-1	South Korea	AY675349	AY675353	221
Soochong	SC-2	South Korea	AY675350	DQ056293	222
Soochong	SC-3	South Korea	AY675351	DQ056294	223
Soochong	SC-4	South Korea	AY675352	DQ056295	224
THAIV	Nakhon Ratchasima/Bi0017/2004	Thailand	AM397664		225

Figure S1. Geographic distribution of DOBV (in blue), SEOV (in red) and HTNV (in green) variants used in this study. Numbers correspond to the codes assigned to each strain in Table S1. The green and red circles indicate places where documented reassortments between SEOV and HTNV strains occur.

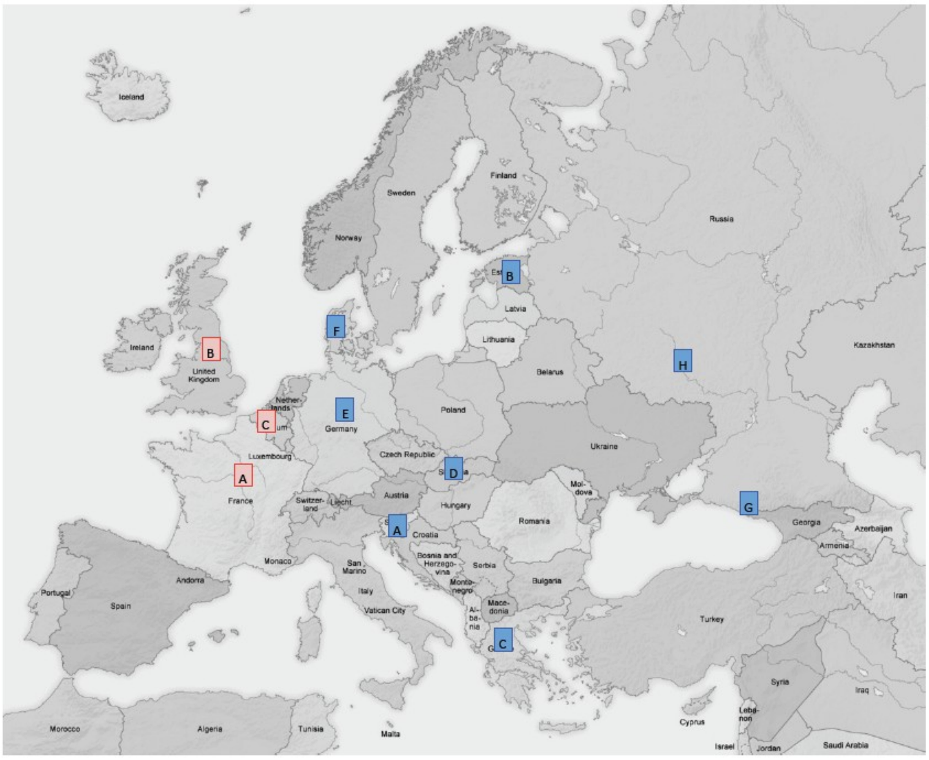

	DOBV
A	17, 45
B	23, 42
C	24, 25
D	26-31, 44
E	32-39
F	43
G	19-22
H	18, 40, 41

	SEOV
A	169
B	124, 152, 153
C	186

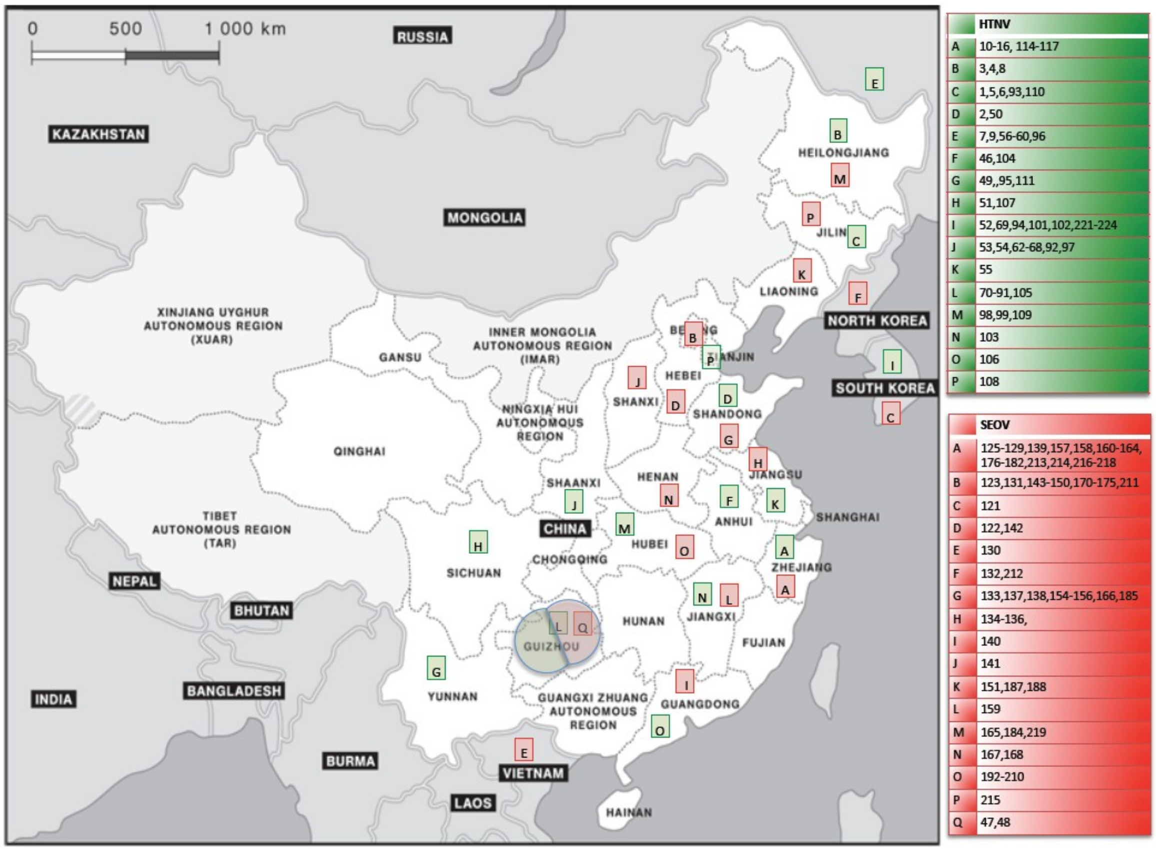

	HTNV
A	10-16, 114-117
B	3,4,8
C	1,5,6,93,110
D	2,50
E	7,9,56-60,96
F	46,104
G	49,,95,111
H	51,107
I	52,69,94,101,102,221-224
J	53,54,62-68,92,97
K	55
L	70-91,105
M	98,99,109
N	103
O	106
P	108

	SEOV
A	125-129,139,157,158,160-164, 176-182,213,214,216-218
B	123,131,143-150,170-175,211
C	121
D	122,142
E	130
F	132,212
G	133,137,138,154-156,166,185
H	134-136,
I	140
J	141
K	151,187,188
L	159
M	165,184,219
N	167,168
O	192-210
P	215
Q	47,48

Figure S2. BEAST time calibrated (ultrametric) trees based on S (**a**) and M (**b**) segments. Root ages were arbitrarily assigned to 1. Nodes before the threshold are considered as species diversification events, whereas branches crossing the threshold define clusters following a coalescent process (in color). Inserts illustrate the Lineage-through-time (LTT) plots. The position of the switches between two evolution patterns (red line) were determined by the method of Pons *et al*.

a

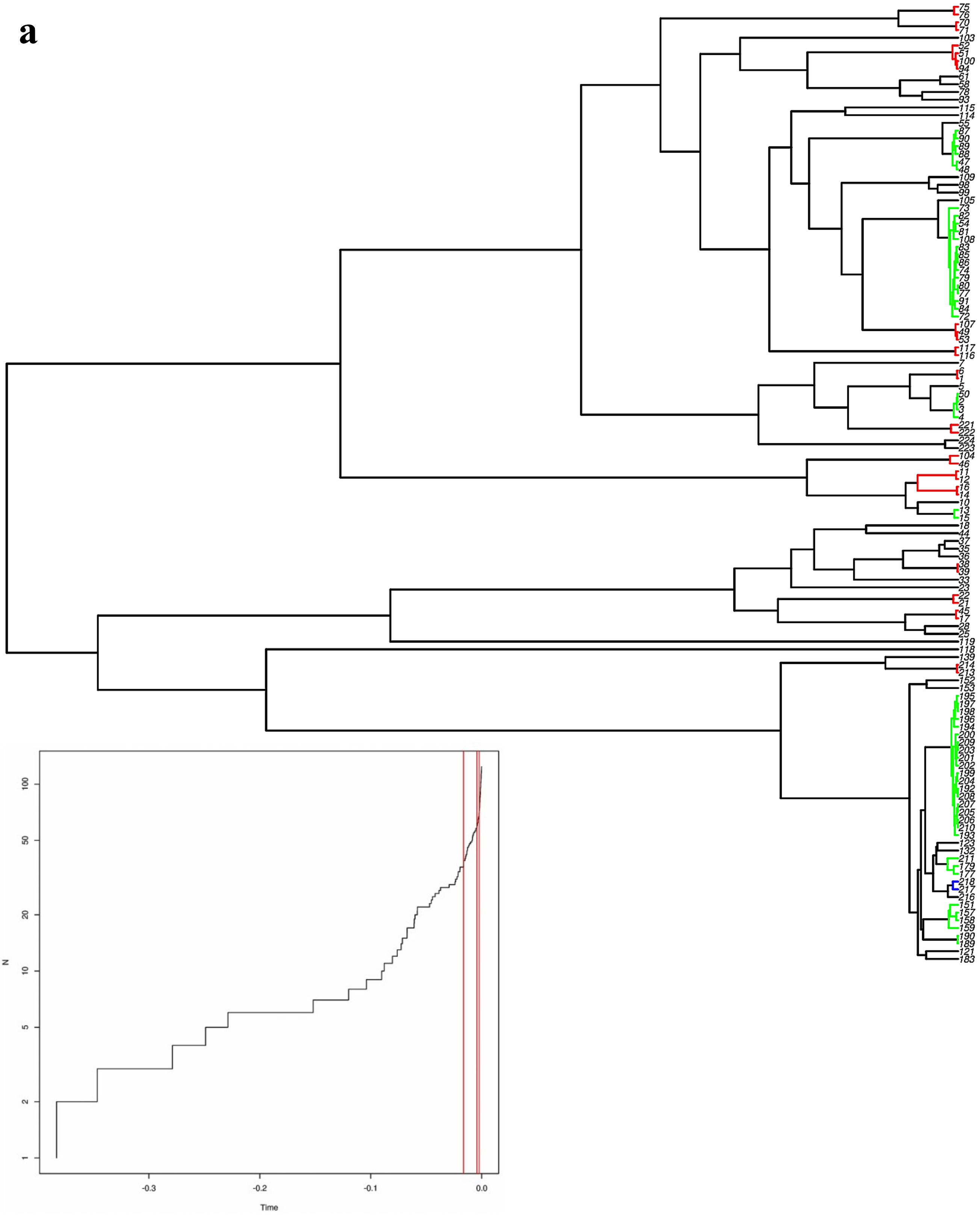

Figure S2. *Cont.*

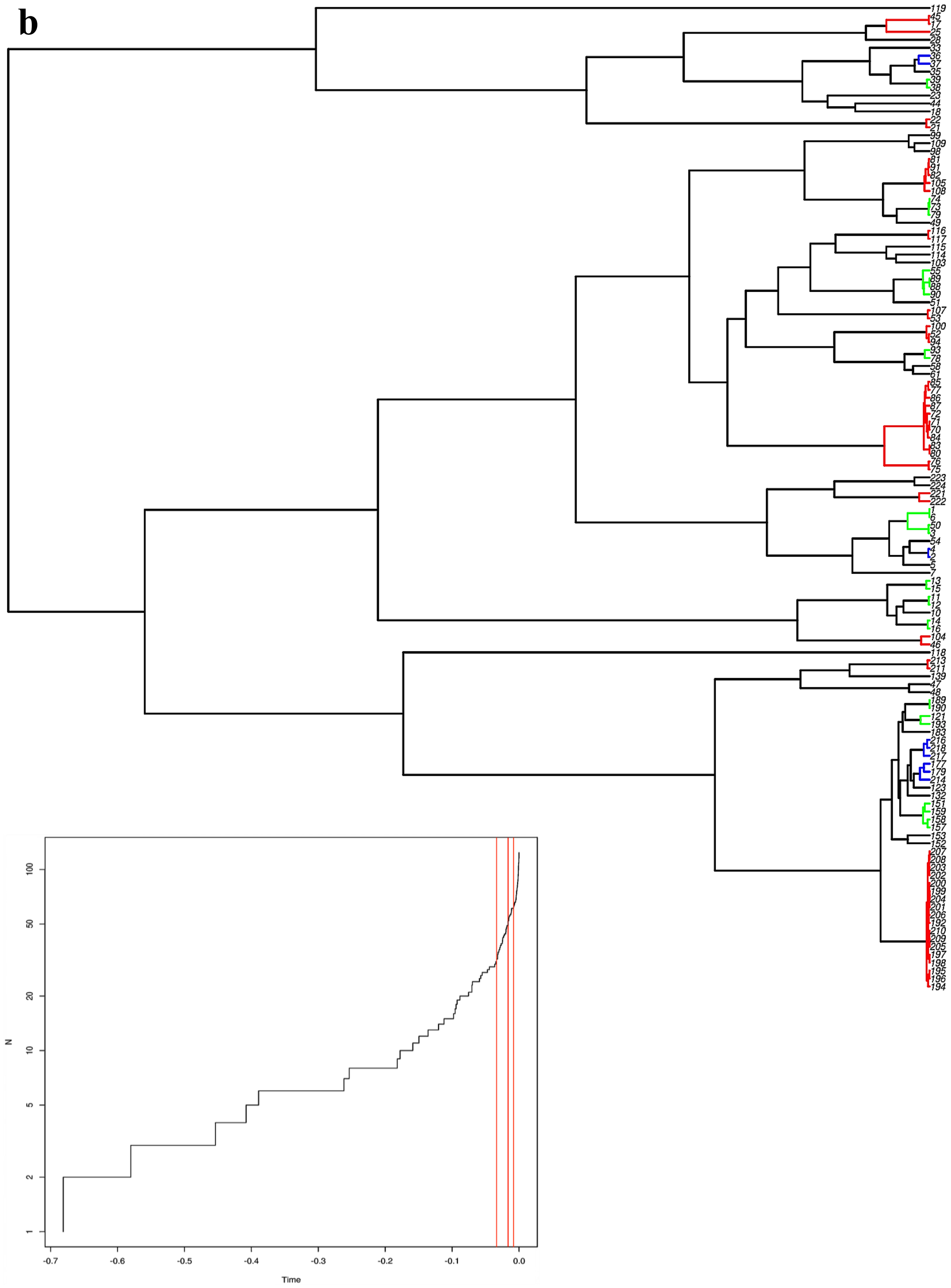

Table S2. List of significant phylotypes. Pi, identifier of phylotype root (referring to identifiers on Figure 4), Cov, coverage (*i.e.*, percentage of taxa annotated with Character state that belongs to the phylotype); Sz, size with *p*-values (in parenthesis) given as fractions, where the denominator indicates the number of shuffles.

Optimization	dataset	Pi	Character state	Cov %	Sz (*p*-value)
DELTRAN	S segment	55	Guizhou	80	20 (0/1000)
		210	Hubei	86	19 (0/1000)
		3	Zhejiang	33	7 (7/1000)
		194	Zhejiang	24	7 (47/1000)
		146	Germany	100	6 (0/1000)
	M segment	51	Guizhou	92	23 (0/1000)
		212	Hubei	82	18 (0/1000)
		179	Zhejiang	38	8 (3/1000)
		34	Zhejiang	33	7 (5/1000)
		13	Germany	100	6 (0/1000)
	S segment (large dataset)	393	Beijing	41	7 (0/1000)
		423	Beijing	41	7 (0/1000)
		104	Guizhou	24	6 (3/1000)
		151	Guizhou	48	12 (0/1000)
		319	Hubei	86	19 (0/1000)
		123	Shaanxi	73	8 (0/1000)
		318	Zhejiang	50	18 (0/1000)
		6	Zhejiang	19	7 (20/1000)
		222	Germany	100	8 (0/1000)
		22	Korea	75	9 (0/1000)
		211	Slovakia	86	6 (0/1000)
ACCTRAN	S segment	55	Guizhou	80	20 (0/1000)
		210	Hubei	86	19 (0/1000)
		3	Zhejiang	33	7 (28/1000)
		178	Zhejiang	33	7 (28/1000)
		146	Germany	100	6 (0/1000)
	M segment	51	Guizhou	92	23 (0/1000)
		174	Hubei	86	19 (0/1000)
		190	Zhejiang	29	6 (42/1000)
		34	Zhejiang	33	7 (5/1000)
		13	Germany	100	6 (0/1000)
	S segment (large dataset)	393	Beijing	41	7 (0/1000)
		423	Beijing	41	7 (0/1000)
		104	Guizhou	24	6 (19/1000)
		151	Guizhou	48	12 (0/1000)
		319	Hubei	86	19 (0/1000)
		123	Shaanxi	73	8 (0/1000)
		6	Zhejiang	19	7 (50/1000)
		252	Zhejiang	50	18 (0/1000)
		222	Germany	100	8 (0/1000)
		22	Korea	75	9 (0/1000)
		211	Slovakia	86	6 (0/1000)
